# The diet of the striped hyena in Nepal's lowland regions

**DOI:** 10.1002/ece3.6223

**Published:** 2020-04-03

**Authors:** Shivish Bhandari, Craig Morley, Achyut Aryal, Uttam Babu Shrestha

**Affiliations:** ^1^ Himalayan Biodiversity Network Nepal Bharatpur Nepal; ^2^ Department of Forest and Resource Management Toi Ohomai Institute of Technology Rotorua New Zealand; ^3^ Global Institute for Interdisciplinary Studies Kathmandu Nepal; ^4^ Faculty of Science School of Life and Environmental Sciences Charles Perkins Centre The University of Sydney Sydney NSW Australia; ^5^ Save Dot International Limited Auckland New Zealand; ^6^ Institute for Life Sciences and the Environment University of Southern Queensland Toowoomba Qld Australia

**Keywords:** diet, *Hyaena hyaena*, Nepal, predators, wildlife

## Abstract

Striped hyenas (*Hyaena hyaena*) are extremely rare in Nepal, and only a few people have studied them in their natural forest and grassland habitat. Their rarity is due to anthropogenic pressures such as hunting, habitat modification, being killed on roads, and depletion of their natural prey. Here, we studied the feeding ecology of hyenas in lowland, Nepal. We employed an opportunistic sampling to collect hyena scats in a range of habitats and the line transect sampling to identify the prey of the hyena in the study site. We collected 68 hyena scats between 2015 and 2018. Most of the hyena scat (39.7%) was found in the Churia Hill forest followed by riverbed (26.4%), mixed forest (14.7%), Sal (*Shorea robusta*)‐dominated forest (11.7%), and grassland area (7.3%). We found eleven mammalian prey species, plants, and some unidentified items in the hyena scats. The frequency of occurrence and relative biomass of the medium‐sized wild boar (*Sus scrofa*) were higher than other smaller prey species such as hare (*Lepus nigricollis*) and rhesus macaque (*Macaca mulatta*). Similarly, the proportion of large prey species such as nilgai (*Boselaphus tragocamelus*) in the hyena diet was lower compared with wild boar, hares, and rhesus macaques indicating medium‐sized wild boar is the most preferred prey species. Livestock contributed 17.3% of the total dietary biomass. Domesticated species such as goats, sheep, cows, and even dogs were found in the diet of hyenas. Predation of livestock by hyenas could cause conflict, especially if this ongoing issue continues in the future. Rather, more conservation effort is required in lowland areas of Nepal to protect the hyenas' natural prey species, particularly in wildlife habitats to reduce the lure of taking domestic livestock. Similarly, conservation education at the local level and active involvement of government authorities in the conservation of this species might be helpful to mitigate human–hyena conflict in the human‐dominated landscape.

## Graphical abstract

We employed scat‐sampling method to understand the prey diversity in the hyenas' diet and found eleven mammalian prey species, plants, and some unidentified items in the hyena's scat. Livestock contributed 17.3% of the total biomass. In addition, hyenas have positive selection towards livestock such as dogs and predation of livestock by the hyenas could cause conflict between human and hyena.

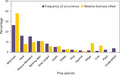

## 1 INTRODUCTION

The conflict between people and large predators is a burgeoning conservation issue worldwide (Inskip & Zimmermann, 2009; Treves & Karanth, 2003), especially as people are expanding into habitats once the domain of wild animals (Tollefson, 2019). With this expansion, new roads are being built, fences erected, and pristine forests felled for agriculture throughout the world, which then exposes wildlife to conflict situations with humans (Kleinschroth, Healey, Gourlet‐Fleury, Mortier, & Stoica, 2017; Laurance & Arrea, 2017). Exposure to humans is even greater with large predators, as they roam large territories in search of prey (Linnell, Swenson, & Andersen, 2001; Singh, Gopanaswamy, & Karanth, 2010), and can kill farmed livestock (Mondal, Sankar, & Qureshi, 2012; Wegge, Odden, Pokharel, & Storaas, 2009). In response, farmers may retaliate for livestock losses by killing wild predators (Aryal, Brunton, Ji, Barraclough, & Raubenheimer, 2014; Valeix, Hemson, Loveridge, Mills, & Macdonald, 2012). These factors, in combination, further exacerbate the decline of many large predators (Linnell et al., 2001; Woodroffe, 2000). Furthermore, the decline is not always uniform as most large predators live in protected parks, while those that reside outside of refuge areas are often persecuted and killed (Linnell et al., 2001). Unfortunately, resources to mitigate potential human–carnivore conflict are limited, which has further contributed to the decline of these large predators (Broekhuis, Cushman, & Elliot, 2017).

In Nepal, most conflict with predators occurs in the surrounding human‐dominated landscape bordering protected areas as farmers are converting wildlife areas into agricultural land and may lose livestock (Aryal, Brunton, Ji, et al., 2014; Bhattarai & Fischer, 2014). However, this human‐dominated landscape also plays a vital role in conserving species, as some communities spend considerable effort to manage these areas because Bengal tigers (*Panthera tigris*), common leopards (*Panthera pardus*), wild dogs (*Cuon alpines*), and striped hyenas (*Hyaena hyaena*) are still present. The farmers in this landscape rely heavily on natural forests to graze livestock, cut grass, grow crops, gather foliage to provide food for their animals, and collect firewood.

The striped hyena exists in the lowland human‐dominated landscape of Nepal (Baral & Shah, 2008) but its numbers are falling, mainly due to anthropogenic causes (Hofer & Mills, 1998; AbiSaid & Dloniak, 2015; Creel et al., 2018). The natural distribution of the striped hyena ranges from northern and eastern Africa to Arabia and surrounding countries, western and southern Asia, including Nepal (Alam, Khan, & Pathak, 2015; Frembgen, 1998; Hofer & Mills, 1998; Kasparek, Kasparek, Gozcelioglu, Colak, & Yigit, 2004; AbiSaid & Dloniak, 2015). Hyenas in all these areas face multiple threats from habitat loss, retaliatory killing, persecution, poisoning, vehicles, and hunting them for meat that is used for medicinal purposes (Bhandari & Chalise, 2016; Hofer & Mills, 1998; Kolowski & Holekamp, 2006; Tourani, Moqanaki, & Kiabi, 2012). Another contributing factor for their decline is because their prey is also being depleted (Alam et al., 2015; Bhandari, Rijal, & Khanal, 2015; Hofer & Mills, 1998; Mondal et al., 2012; Qarqaz, Baker, & Amr, 2004). The ever‐increasing encroachment of people has also caused the distribution of hyenas to decline markedly in other countries such as Armenia and Turkey (Hofer & Mills, 1998; Kasparek et al., 2004; Khorozyan, Malkhasyan, & Murtskhvaladze, 2011; Kruuk, 1976).

Widespread road construction and infrastructure development in this human‐dominated landscape have further exposed hyenas to conflict with people (Carter et al., 2013; Joshi et al., 2016). Hyenas are occasionally struck by vehicles when they are scavenging or crossing roads in Nepal (Adhikari et al., 2018). About 20–30 hyenas are killed each year by vehicles colliding with hyenas in Israel (Hofer & Mills, 1998); however, the exact number in Nepal is unknown. Another source of conflict is that people hunt wild animals which are also prey of hyena, often illegally, for food such as chital (*Axis axis*), sambar (*Rusa unicolor*), wild boar (*Sus scrofa*) and the northern Indian hare (*Lepus nigricollis*). Collectively, these direct and indirect anthropogenic pressures place enormous stress on wild populations of large predators, threatening their very survival (Athreya, Odden, Linnell, Krishnaswamy, & Karanth, 2013; Carter et al., 2013; Jnawali et al., 2011; Joshi et al., 2016).

The estimated population of hyenas in Nepal is <100 individuals (Hofer & Mills, 1998; Jnawali et al., 2011); however, a recent survey in the mountainous areas of Western Nepal found a population not previously recorded, thus, indicating their distribution requires further study (Bhandari & Bhusal, 2017). In Nepal, hyenas are reported in small isolated populations in tropical and subtropical National Parks, such as Parsa, Chitwan, Banke, Bardia, and Suklaphanta, and also in the surrounding human‐dominated areas near these parks (Adhikari et al., 2018; Bhandari & Chalise, 2016; Bhandari et al., 2015; Jnawali et al., 2011). Thus, we only have a limited understanding of their true distribution and range in Nepal.

We also have little information on the relationship between people and hyenas in Nepal, primarily due to the nature of hyenas as they are secretive and because they are only ever observed in small isolated populations. However, if people do encounter hyenas it is usually when farmers kill hyenas in retaliation for losing their livestock or if they see hyenas scavenging (Bhandari & Chalise, 2016; Hofer & Mills, 1998). One method to understand hyena–people conflict is to examine what hyenas eat and whether this includes any farmed livestock. Analyzing scat contents is a noninvasive technique used to study the food habits of predators (e.g., Andheria, Karanth, & Kumar, 2007; Aryal, Brunton, McCarthy, et al., 2014; Karanth & Sunquist, 1995; Koirala et al., 2012; Mukherjee, Goyal, & Chellam, 1994; Stoen & Wegge, 1996). From scat records, we can determine what hyenas consume (Stoen & Wegge, 1996; Wagner, 2006; Wegge et al., 2009). Understanding the contents of a hyena scat can also divulge information about their life‐history strategies, prey preference, and geographical distribution which can be used to improve conservation management strategies (Hayward, Jedrzejewski, & Jedrzejewska, 2012; Selvan, Veeraswami, Lyngdoh, Habib, & Hussain, 2013; Sunquist & Sunquist, 1989). It is also useful to understand the importance of different prey types in their diet, especially if their prey species is also declining and/or hunted by people (Hofer & Mills, 1998; Jnawali et al., 2011). Only scant information on the diet of hyenas in Nepal is known, but main food sources are thought to be medium‐sized prey such as wild boar (38 kg) and barking deer (*Muntiacus muntjak* (20 kg) (Alam & Khan, 2015; Hofer & Mills, 1998; Wagner, 2006). Hyenas may also take small species like rhesus macaques (*Macaca mulatta*) (8 kg) right up to larger‐sized prey species such as sambar (125 kg). Complicating the information on hyena diet is that hardly any information exists on the distribution and availability of prey species in lowland Nepal.

We studied the scats of the striped hyena in several lowland habitats of Nepal, which included natural forests, steep mountains, and the human‐modified grassland and valley. In addition to the scat analyses, we examined variations in prey availability for hyenas across these different habitats. With the expansion of people into more natural areas, concerns have been expressed that hyenas could switch from their natural prey to eating domestic prey. While human‐dominated areas have a different assemblage of animals (mainly domestic livestock) from that of the natural forests, we asked whether the diet of the striped hyena was similar for all habitat types. Thus, our goal is to ascertain if hyenas are eating domestic livestock in these highly modified lowland areas of Nepal.

## 2 MATERIALS AND METHODS

### 2.1 Study area

**FIGURE 1:**
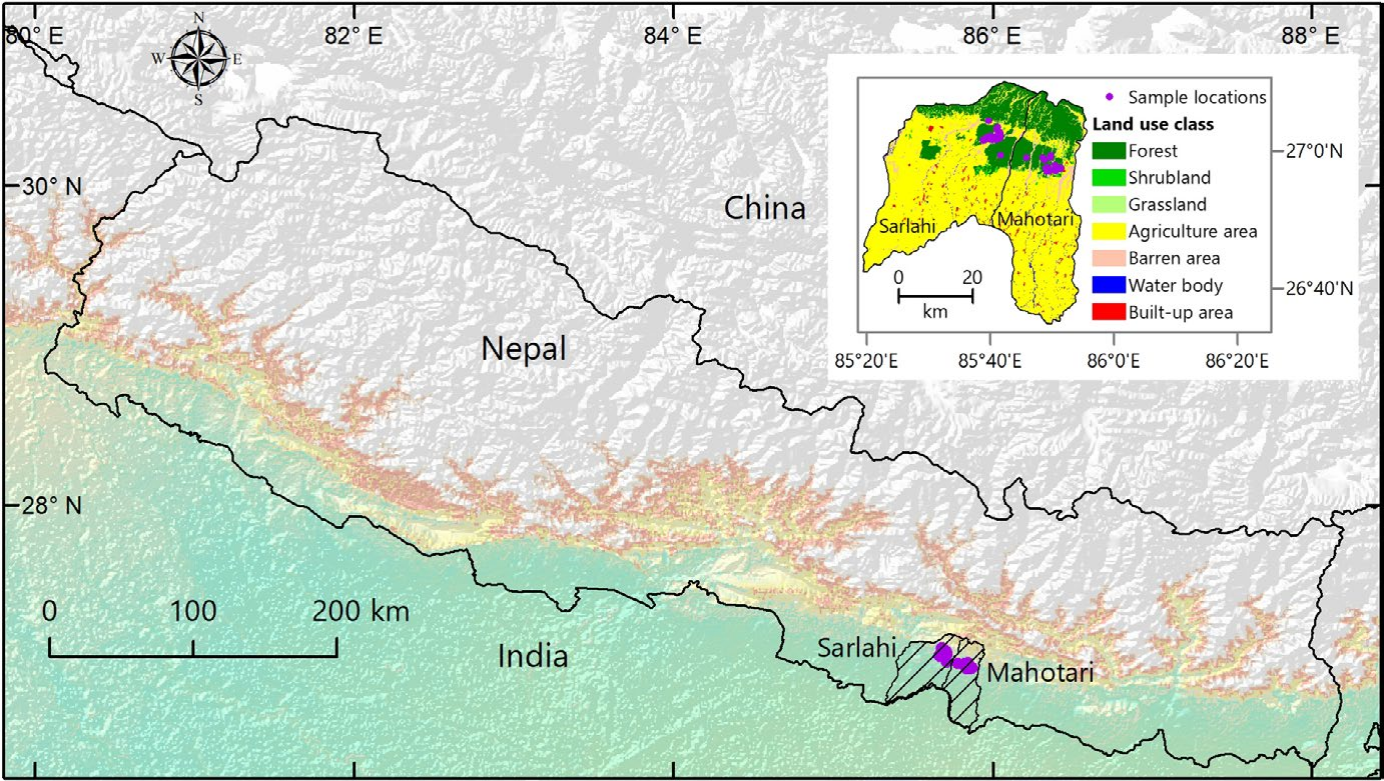
The study site locations, the Mahottari and Sarlahi District in lowland Nepal. The inset map shows the land use maps of two studied districts and sample locations

This study was carried out in two lowland districts (Sarlahi and Mahotari) of Nepal which comprised of tropical forest, dominated by Sal (*Shorea robusta*), a mixed forest of Eucalyptus (*Eucalyptus spp*.), Khair (*Acacia catechu*), and sissoo (*Dalbergia sissoo*) (Figure 1). The study area was located between 26.98–27.10 °N latitude and 85.67–85.87 °E longitude and was approximately 350 km^2^ (Figure 1). The average temperature for this area varies from 12°C in winter to 30°C in summer (DFRS, 2014). This area is home to more than 20 mammal species, 150 species of birds, and 40 species of reptiles and amphibians (Bhandari et al., 2015; Chettri & Chhetry, 2013; Shrestha, 2003). Moreover, large mammals, like the Asian elephant (*Elephas maximus*), also use this area as a major corridor (Smith & Mishra, 1992). About 1,500 people reside in the area growing crops such as corn (*Zea mays*), wheat (*Triticum aestivum*), potato (*Solanum tuberosum*), rice (*Oryza sativa*), and farm livestock such as cow (*Bos taurus indicus*), buffalo (*Bubalus arnee*), goats (*Capra hircus*), and domestic pig and poultry which are a major economic resource for the local people.

### 2.2 Scat identification, collection, and analysis

We used the protocol devised by Aryal, Hopkins, Ji, Raubenheimer, and Brunton (2012), Aryal, Brunton, McCarthy, et al. (2014) and Koirala et al. (2012) to ascertain what prey was eaten by hyenas. The field surveys for scats and observations of prey availability were carried out from November to December 2015, February to April 2016, December 2017, and April to June 2018. Based on the information provided by the local people about the potential territories of hyenas as they sight hyenas, we collected scats at random from the following habitats (e.g., the Churia Hill ranges, mixed forest, riverbeds, and grasslands). Our sampling effort was equal in all habitats. Hyena scats were distinguished from the other predators by their shape and size (Bhandari et al., 2015; Sharma, Jhala, & Sawarkar, 2005). We also used other associated signs, for example, pugmarks to help identify if the scats were from hyenas. We omitted any scats from our data if we could not confirm it was a hyena scat.

The collected scats were stored in zip‐locked bags, labeled, and then sun‐dried for a day. Each scat was assumed to be an independent sample. We washed the scats through a sieve (1.5 mm) under running water and macroscopically sorted the prey remains such as hair, teeth, claws, and bones as described by Oli, Taylor, and Rogers (1993), Karanth and Sunquist (1995), Stoen and Wegge (1996), and Ramakrishnan, Coss, & Pelkey, (1999). To identify the prey species in each scat, a total of 20 hairs were randomly picked and dissolved into a mixture of ethyl alcohol and diethyl ether (1:1) solution in a petri dish for 30 minutes. After selecting these 20 hairs, five hairs were randomly selected and laid out in parallel lines on a slide that was then painted with transparent nail polish to observe the cuticle pattern of the hairs. The prepared slides were dried for 1 hr at room temperature. After removing the hairs from the slide, the hair imprint on the slide was observed though a compound stereoscopic microscope under 400× magnification. Another five hairs from our original sample of 20 were placed into acetone for 45 min. We used the same procedure above to observe the medullar pattern of the hair (Aryal et al., 2012; Koirala et al., 2012). The recorded cuticular and medullary images of the hairs were then compared by using a reference key produced by DeMarinis and Asprea (2006) and Thapa (2013). We also used reference images prepared from the National Trust for Nature Conservation to confirm our findings (Bhandari, Chalise, & Pokharel, 2017).

Floyd, Mech, and Jordan (1978) described the regression equation (*Y* = 0.38 + 0.02*X*) to analyze captive wolf scats. We followed the same guidelines used by Floyd et al. (1978) to estimate the biomass of prey consumed by hyenas (*Y*), where *X* = the live weight of prey species. We calculated the relative biomass consumed by the hyenas using the formula:

$$\text{Relative biomass consumed}=\frac{\left(\text{frequency of occurrence}\times Y\right)}{\Sigma \left(\text{frequency of occurrence}\times Y\right)}\times 100.$$

We used Ivlev's electivity index (*E_i_*) to measure the relationship between proportion of prey species found in the scats and prey available in nature, where *E_i_* = (*r_i_* − *P_i_*)/(*r_i_* + *P_i_*). In this formula, *r_i_* represents the relative abundance of a prey in a hyena's diet and *P_i_* is the prey's abundance in nature. *E_i_* = corresponds from −1 (total avoidance) to 1 (high preference).

We tested what type of prey is eaten more frequently, and the difference is statistically significant or not. We performed a Fisher exact test to determine whether we could distinguish between two similar species within a single hyena scat. The *p*‐value indicated that one species occurred significantly more often in the scats than the other species.

### 2.3 Prey availability analysis

Following (Anderson, Laake, Crain, & Burnham, 1979; Burnham, Anderson, & Laake, 1980), we walked a total 93 random transects during the daytime (average transect length 1.8 km; range 1.4–2.6 km) to observe the potential available prey for hyenas. While the transect lengths varied, the search effort (walking speed and pace) was the same for all transects, so we could concentrate on seeing even the smallest species present. The transects were randomly laid out within five major habitats: Churia Hill forest, riverbed, mixed forest, Sal‐dominated forest, and grassland (Table 1). The estimated encounter rate index along the surveys was calculated for each prey species as encounter rate = number of sightings/total distance travelled.

**TABLE 1:** Description of the habitat types along transects

Habitat type	Description
Churia Hill forest (CHF)	This forest is dominated by *Shorea robusta, Skimmia arborescens, Terminalia alata,* and *Schima wallichii* including tall and short grasses. CHF also contains seasonal rivers
Riverbed (RB)	It lies along the riverside along with *Acacia catechu* forests and comprises small patches of sand, gravel or stone, grass, and shrubs
Mixed forest (MF)	Temperate, deciduous and hardwood forests, such as *Adina cordifolia*, *Schima wallichii*, *Dalbergia sissoo*, *Dillenia pentagyna*, *Bombax ceiba*, and *Albizia* spp.
Sal‐dominated forest (SDF)	Forest dominated by *Shorea robusta* with an admixture of *Terminalia* spp.
Grassland area (GA)	Small patches of grassland with shrubs near to the edge of agricultural land, river, or forests

The mean live body mass of the prey species was taken from published data (Bhandari et al., 2017; Bhattarai & Kindlmann, 2012; Wegge et al., 2009). We used Shannon's diversity index (*H*) to measure the abundance and evenness of the prey within the different habitats. We used the formula, *H* = −Σ *p_i_* (ln *p_i_*), where p_i_ = proportion of each species in the sample. High values of *H* would be representative of more diverse communities. Shannon's equitability (EH) was calculated by dividing *H* by ln *S*, where ln *S* was the total number of species found in the habitat. Values nearing or equal to 1 represented complete evenness while values near or equal to 0 showed no evenness.

## 3 RESULTS

Most scats (39.7%) were found in the Churia Hill forest followed by the riverbeds (26.4%), mixed forest (14.7%), Sal‐dominated forest (11.7%), and grassland areas (7.3%). The deposition of scats across habitat types were significantly different (ANOVA *f* = 6.00, *df *= 19; *p* = .004). We recorded 33 scats (49%) with two discrete prey species, 14 scats (21%) with only one prey item, 13 scats (19%) with three prey species, 5 scats (7%) with four prey species, and 3 scats (4%) containing five or more prey species. Thus, our results indicate that hyenas eat a variety of prey.

**FIGURE 2:**
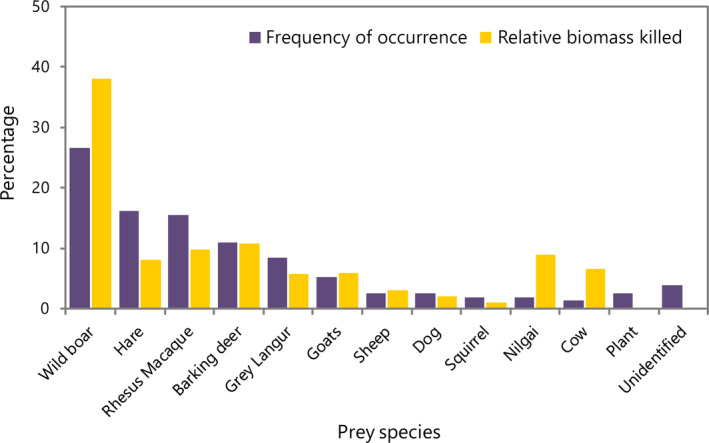
Percentages of frequency occurrence and relative biomass of prey species found in the hyena scats

Within the 68 scats examined, eleven different mammalian prey species were recorded. Plant material was also found in the hyena scats along with the remains of some birds and insects although this was not examined in any detail as the proportion found was negligible (Figure 2). Overall, the most frequently eaten prey with the greatest relative biomass was wild boar (Fisher exact test; *p* = <.05). Other mammalian prey species found in the hyena scats were hares (*p *= <.05), rhesus macaque (*p *= <.05), barking deer (*p *= >.05), grey langur (*p *= <.05), goats (*p *= >.05), domestic sheep (*Ovis aries*) (*p *= <.05), domestic dogs (*Canis familiaris*) (*p *= <.05), squirrels (*Funambulus palmarum*) (*p *= <.05), nilgai (*Boselaphus tragocamelus*) (*p *= >.05), and cow (*p *= <.05).

While both wild and domesticated species were found in the scats, the frequency of occurrence for wild prey was 81.8% while only 11.7% was domestic farm animals. Similarly, the relative biomass of wild versus domestic farm animals was 82.6% for the wild prey and 17.4% for domestic prey (Figure 2).

The Ivlev's electivity index indicated that smaller hares, domestic dogs, and squirrels were also eaten by hyenas, even more than the larger prey such as wild boar, barking deer, Grey langur, and rhesus macaque. Although Chital, sambar, jungle fowl (*Gallus gallus*), and porcupine (*Hystrix indica*) were frequently observed in our field surveys, we found no scat remnants of these species (Table 2).

**TABLE 2:** Hyena prey consumed and available in lowland, Nepal, where a = % of prey occurrence found in the hyena scats and b = % of prey proportion observed along our transects (*N* = 93)

Species	Prey use^a^	Prey available^b^	Ei
Wild boar (*Sus scrofa*)	26.62	10.95	0.4
Hare (*Lepus nigricollis*)	16.23	0	1
Rhesus macaque (*Macaca mulatta*)	15.58	27.03	−0.2
Barking deer (*Muntiacus muntjak*)	11.03	1.26	0.7
Grey langur (*Semnopithecus hector*)	8.44	10.52	0.7
Goats (*Capra aegagrus*)	5.19	9.63	−0.29
Sheep (*Ovis aries*)	2.59	2.92	−0.05
Dog (*Canis familiaris*)	2.59	0	1
Squirrel (*Funambulus palmarum*)	1.94	0	1
Nilgai (*Boselaphus tragocamelus*)	1.94	5.05	−0.44
Cow (*Bos spp*.)	1.29	20.36	−0.88
Chital (*Axis axis*)	0	3.5	−1
Sambar (*Rusa unicolor*)	0	0.68	−1
Jungle fowl (*Gallus gallus*)	0	6.62	−1
Porcupine (*Hystrix indica*)	0	1.46	−1
Plant reminants	2.59	NA	NA
Unidentified	3.89	NA	NA

**FIGURE 3:**
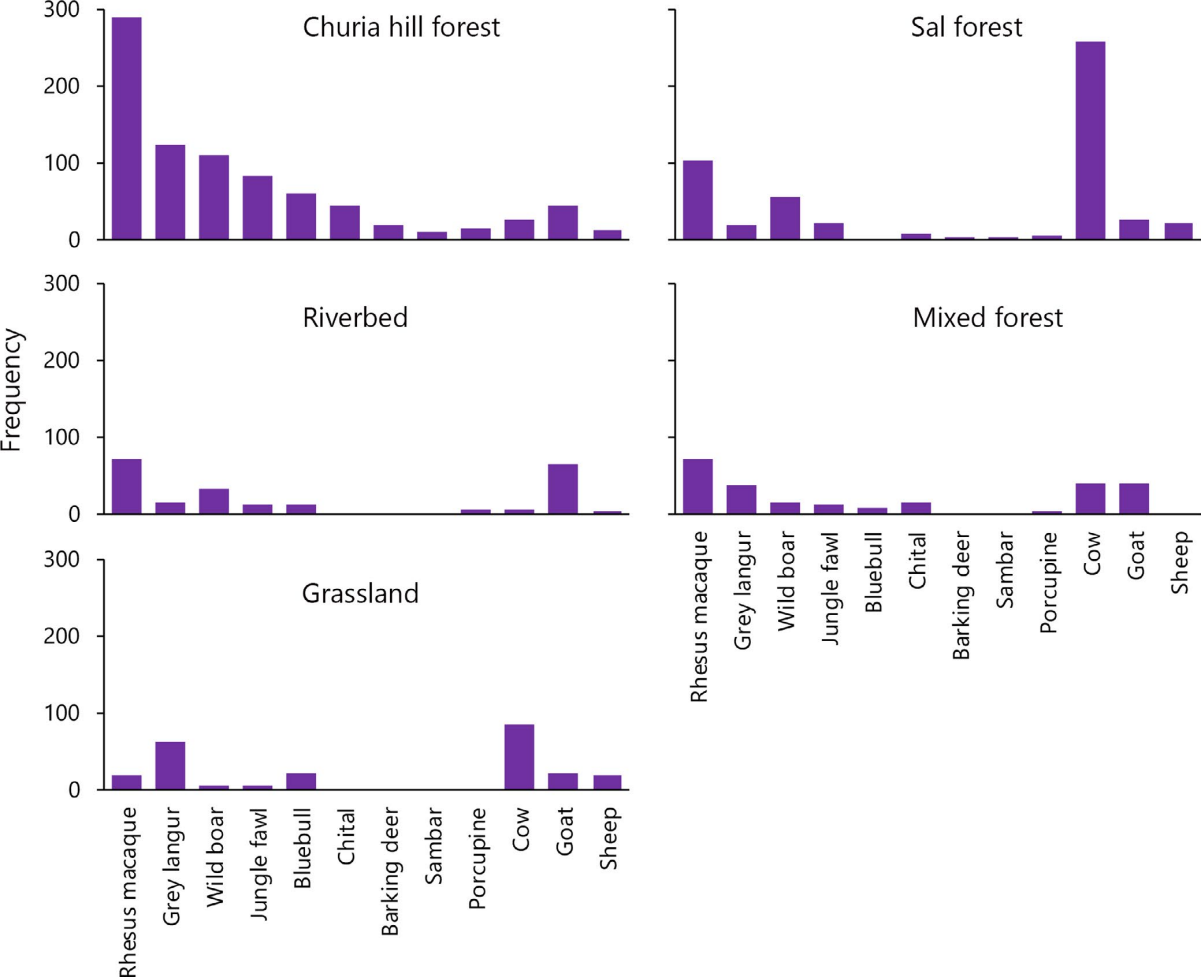
Frequency of prey species found in different habitat types in the study site

We counted a total of 2,053 potential individual prey (Table 3). There were significant differences between the distribution of prey species between habitats (Churia Hill forest, riverbed, mixed forest, Sal‐dominated forest, and grassland area) (*f* = 8.02, *df *= 92, *p *= <.05). Livestock (e.g., cows and goats) were observed in all habitats (Figure 3). Rhesus macaques were the most frequently observed animal followed by cows and wild boar, but this was probably an artifact of curiousness and mobility. We saw no hares, dogs, or squirrels even though they were found in the scats. Further, we saw few sambar, porcupines, and barking deer (Table 2). In terms of prey availability, the Churia Hill forest was the most diverse community (Shannon's diversity index *H* = 2.1) followed by the riverbeds (*H* = 1.88), Sal‐dominated forest (*H* = 1.71), and mixed forest (*H* = 1.5). Species evenness (EH) was highest in the grassland areas (EH = 0.85), followed by Churia Hill forest (EH = 0.81) and the riverbeds (EH = 0.78). Species evenness (EH) in the mixed forest and Sal‐dominated forest was 0.68.

**TABLE 3:** Encounter rate estimation of hyenas major prey from the line transect sampling

Species (weight in kilogram)	Sample size	Encounter rate (km^−2^)
Chital (45)	69	0.3
Wild boar (38)	225	1.2
Sambar (125)	14	0.07
Bluebull (169)	105	0.5
Jungle fowl (2)	136	0.7
Rhesus monkey (6)	555	2.9
Grey Langur (8)	216	1.16
Porcupine (13)	30	0.1
Goat (26)	199	1.0
Sheep (27)	60	0.3
Cow (180)	418	2.2
Barking deer (20)	26	0.1

## 4 DISCUSSION

Our study presents an insight into the diet of the striped hyenas in the human‐dominated lowland areas of Nepal. In all, eleven mammalian prey species were identified in the hyena scats collected. Similarly, Mondal et al. (2012) in India found nine different mammalian prey from 86 striped hyena scats. Our study showed that hyenas consume a variety of vertebrate prey from small squirrels (1.5 kg) through to large prey like nilgai (169 kg). However, medium‐sized wild boars (38 kg) were the most common prey species eaten by hyenas, followed by hares (2.5 kg) and rhesus macaques (6 kg). While hares were regularly found in the hyena scats, no hares were seen in the encounter transect surveys primarily because they are crepuscular/nocturnal (Chakraborty, Srinivasulu, Jordan, & Bhattacharyya, 2005) and our survey counts were carried out during daylight hours. Conversely, Rhesus macaques, a diurnal animal, were the most commonly observed animal in our encounter surveys.

In terms of the relative biomass consumed, these lowland hyenas ate more small and medium‐sized prey species such as wild boar (around 38 kg). Striped hyenas in Africa and India also ate more medium‐sized prey species (Alam & Khan, 2015; Wagner, 2006). In contrast, the striped hyenas within the Sariska Tigers Reserve in Western India ate more large prey species, such as nilgai and chital (Mondal et al., 2012). While large nilgai and chital were easier to see in our encounter surveys than the medium‐sized wild boar, only a few of these ungulates were observed compared to the more mobile and gregarious wild boars. Wild boars are widely distributed throughout Nepal and are equally at home in the human‐dominated areas as well as the protected natural forests (Karki, 2011). Wild boars are also hunted by other large predators such as tigers and leopards so the hyenas face considerable competition for wild boar (Aryal, Lamsal, Ji, & Raubenheimer, 2016; Wegge et al., 2009). Nevertheless, Alam and Khan (2015) found that wild boar constituted a large part of the diet of hyenas in the Gir National Park and Sanctuary, India. Thus, wild boars are considered a key prey species for hyenas.

Differences in habitat influenced prey selection for the hyenas. The proportion of wild boar in the diet of hyenas is an artifact of their general availability, whereas species like sambar and barking deer, who are restricted to the most protected areas of Nepal (Karki, 2011), were disproportionately represented in the diet of lowland hyenas. The Churia Hill forest in this area, a mountain range of the outer Himalayas, was the least disturbed habitat, whereas the mixed forest habitat and the grasslands, particularly in the valleys, have been severely fragmented and modified for crop cultivation and livestock farming. The open‐braided riverbeds provide easy open access for the hyenas to move quickly from one location to another. While the sampling effort was equal for each habitat, we found considerably more scats in the Churia Hill forests. The Churia Hill forest is a semi‐arid environment that has dense stands of forest which provides plenty of natural food and shelter for a wide range of potential prey (Thapa, 2011). We also encountered the highest diversity of species within this habitat that supports the results found by Karki (2011). Few people reside in the Churia Hill forests compared to the other habitats because of its typography, short crop‐growing season, and climate. Wild animals roam unencumbered from fences, roads, and human‐made structures. Consequently, the Churia Hill forest provides good shelter for both hyenas and their prey rather than the modified more accessible human‐dominated mixed forests and grassland areas.

Previous studies, for example, Sunquist and Sunquist, (1989); Kruuk, (1976); Wagner, (2006) Trinkel, (2010), reported that the hunting behavior of predators depends on prey size and species density and that hyenas would hunt large prey if circumstances permitted (Kruuk, 1976; Wagner, 2006). Even though large prey could be killed by a clan of hyenas, Wagner (2006) observed that most hyenas hunt alone. This solitary hunting behavior could explain why more small‐ to medium‐sized prey were found in the scats of Nepalese hyenas. While large cows and nilgai were frequently observed in the transect surveys, they were only found in less than four percent of the scats, suggesting large prey were rarely hunted by hyenas or if they were consumed, and then, this is possibly a result of hyenas scavenging kills made by other larger predators rather than hyenas killing large prey. It would be uncommon for a solitary hyena to kill a large sambar (125 kg) or nilgai (169 kg) by themselves but a clan could kill one. A single hyena could possibly take a chital (45 kg) if an opportunity arose, although no chital remains were detected. Sharing habitats with large predators like tigers and leopards, which predominately hunt large prey (Aryal, Brunton, Ji, et al., 2014; Stoen & Wegge, 1996; Wegge et al., 2009), may force hyenas to hunt smaller prey species, as we found in their diet. Indeed, in terms of their frequency of occurrence, hares, rhesus macaques, barking deer, and grey langurs were the next largest assembly of prey in the diet of the hyenas.

Hyenas are opportunistic generalist predators and so will often eat more than one prey species, as the majority of the scats had more than two prey species. A small hare, for example, would be insufficient to fulfill a hyena's daily energy requirement, so they will continue searching for secondary prey when an opportunity presents itself. Alam and Khan (2015) considered hyenas in India to have a broad diet as they identified over 40% of the scats to have 1–3 prey species present; however, most scats had 4–7 prey species, most of which were mammals. We found some unidentified fragments within the scats possibly jungle fowls, which we saw in our encounter transects, reptiles, invertebrates (e.g., dipteran larvae, grasshoppers, beetles, or termites), and organic waste. The dipteran larvae were thought to be a result of the hyenas scavenging prey. Unfortunately, we could not separate and distinguish out what these small remains were. Wagner (2006) reported that hyenas also consumed organic waste produced by humans and occasionally rodents that were present in compost and waste deposits. Further study is required to confirm the traces of human organic waste items within the diet of hyenas.

We found domestic animals within the hyena scats. Domestic dog hairs were detected as they are often used by farmers to try and ward off animals from eating their crops. Goats and sheep were also present in the diet. Hyenas have been reported to feed on farmed livestock (Bhandari et al., 2017; Frembgen, 1998; Kasparek et al., 2004; Kruuk, 1976; Wagner, 2006) but in our study, we cannot say for certain if they had been killed by hyenas or they were the remains left by other predators; however, the dipteran larvae seen suggest they were scavenged remains. Alam and Khan (2015) found that domestic prey represented about a fifth of the diet (20.9%) in striped hyenas from Gir National Park Sanctuary in India. There are also reports of attacks by striped hyenas on domestic sheep, goat, and donkeys in North Africa, Israel, Iran, Pakistan, and India; on horses in Iran; and on dogs in India (Alam & Khan, 2015; Hofer & Mills, 1998). Finding livestock in the scats of hyenas is not surprising especially given that people are now modifying wildlife areas into agricultural fields for domestic animals (Joshi et al., 2016; Wegge et al., 2009). People in our study area reported livestock losses and attacks by large predators (Bhandari & Chalise, 2016), but we saw no evidence that linked hyenas to these losses. Nevertheless, some hyenas have been killed in retaliation as two hyenas were poisoned in cage trap set between 2015 and 2016 (Bhandari & Bhusal, 2017).

Our finding of plant matter within the hyena scats is similar to that found by Kruuk (1976) and Mondal et al. (2012) as they detected fruit and vegetables in the scats of hyenas. In the semi‐arid landscape of India, hyenas consumed a lot more vegetable matter as well as mammals, birds, and insects (Alam & Khan, 2015). The plant matter in the diet could have come from plants intermingled with the carcasses of the prey, or another explanation is that the hyenas are raiding crops and eating plants such as corn, melons, and cucumbers. Crop‐raiding behavior is considered a serious problem in countries like Israel (Kruuk, 1976). However, the issue of crop raiding by hyenas was not considered to be a major problem in our field study.

When comparing hyena home ranges (44–72 km^2^) based on studies by Kruuk (1976) and Hofer and Mills (1998), we predict that there may be between 4 and 6 hyenas within our study site. A small population of hyenas such as this would face strong competition with other predators for prey, which may also include livestock. Golden jackals (*Canus aureus*) and leopards are known competitors within our study site while Hofer and Mills (1998) also report that hyenas also compete with wolves (*Canis lupus*) and red foxes (*Vulpes vulpes*) for food in Iran. Interspecific competition for prey may limit hyena numbers in our study site, which is similar to those reported in India and Iran (Alam & Khan, 2015; Hofer & Mills, 1998).

While hyenas in lowland Nepal mainly eat wild prey, finding domestic livestock species within their diet points to a potential source of conflict with people (Aryal, Brunton, Ji, et al., 2014; Bhandari & Chalise, 2016). Unfortunately, even the smallest presence of livestock in the diet of hyenas can provoke negative feelings by farmers, but we did not find any evidence of hyenas directly killing livestock. Indeed, we hypothesize that the domestic prey in the diet was from animals that had either died naturally or were carcasses left by other predators. Hyenas are opportunistic predators and will hunt and scavenge prey when available, including roadkill (Adhikari et al., 2018), and so limiting road development or vehicle speed as is done in many wildlife areas is an option so hyenas are not run‐over (Laurance & Arrea, 2017). Further, preventing forest encroachment and conversion into agriculture is also vital (Tollefson, 2019). We found little evidence of crop raiding during our field study, suggesting it is negligible or nonexistent.

Hyena numbers are already low in the study area and elsewhere in Nepal (Hofer & Mills, 1998; Jnawali et al., 2011), so a conservation action plan agreed to by farmers, conservationists, and local lawmakers are urgently required to protect this native wild carnivore from even further decline. Therefore, we recommend educating people at the local level and providing active involvement of government authorities in the conservation of hyenas. Further, legislating and protecting the remaining natural habitats, along with this their natural prey, is vital so hyenas are not tempted to switch from their natural prey to domestic livestock. If domestic livestock is killed, then compensation in the form of another animal rather than money should be paid to the farmer, and insurance programs to cover livestock and crop loss should be implemented. Finally, while hyenas may not be viewed highly by people (Hofer & Mills, 1998; Kruuk, 1976), their natural role as a scavenger is an important part of Nepal's ecosystem and we should protect them, just like we would if they were tigers, elephants, or leopards. Indeed, their foraging role is indispensable by helping to dispose of diseased, elderly, or infirm animals. Without hyenas, disease transmission is likely and this could ultimately affect many domestic animals.

## CONFLICT OF INTEREST

None declared.

## AUTHOR CONTRIBUTIONS

**Shivish Bhandari:** Conceptualization (equal); data curation (lead); formal analysis (equal); Writing – original draft (equal). **Craig Morley:** Writing – original draft (equal); writing – review and editing (equal). **Achyut Aryal:** Conceptualization (equal); Writing – original draft (equal); Writing – review and editing (equal). **Uttam Babu Shrestha:** Conceptualization (equal); formal analysis (equal); visualization (lead); writing‐original draft (equal); writing – review and editing (equal).

## Data Availability

Raw data: Dryad, https://doi.org/10.5061/dryad.kd51c5b2t
